# Rapid and Accurate Behavioral Health Diagnostic Screening: Initial Validation Study of a Web-Based, Self-Report Tool (the SAGE-SR)

**DOI:** 10.2196/jmir.9428

**Published:** 2018-03-23

**Authors:** Benjamin Brodey, Susan E Purcell, Karen Rhea, Philip Maier, Michael First, Lisa Zweede, Manuela Sinisterra, M Brad Nunn, Marie-Paule Austin, Inger S Brodey

**Affiliations:** ^1^ TeleSage Chapel Hill, NC United States; ^2^ Centerstone Nashville, TN United States; ^3^ Department of Psychiatry Columbia University New York City, NY United States; ^4^ School of Psychiatry University of New South Wales Sydney Australia; ^5^ Department of English and Comparative Literature University of North Carolina at Chapel Hill Chapel Hill, NC United States

**Keywords:** mental health, differential diagnosis, surveys and questionnaires, self-report, primary health care, computer-assisted diagnosis

## Abstract

**Background:**

The Structured Clinical Interview for DSM (SCID) is considered the gold standard assessment for accurate, reliable psychiatric diagnoses; however, because of its length, complexity, and training required, the SCID is rarely used outside of research.

**Objective:**

This paper aims to describe the development and initial validation of a Web-based, self-report screening instrument (the Screening Assessment for Guiding Evaluation-Self-Report, SAGE-SR) based on the Diagnostic and Statistical Manual of Mental Disorders, Fifth Edition (DSM-5) and the SCID-5-Clinician Version (CV) intended to make accurate, broad-based behavioral health diagnostic screening more accessible within clinical care.

**Methods:**

First, study staff drafted approximately 1200 self-report items representing individual granular symptoms in the diagnostic criteria for the 8 primary SCID-CV modules. An expert panel iteratively reviewed, critiqued, and revised items. The resulting items were iteratively administered and revised through 3 rounds of cognitive interviewing with community mental health center participants. In the first 2 rounds, the SCID was also administered to participants to directly compare their Likert self-report and SCID responses. A second expert panel evaluated the final pool of items from cognitive interviewing and criteria in the DSM-5 to construct the SAGE-SR, a computerized adaptive instrument that uses branching logic from a screener section to administer appropriate follow-up questions to refine the differential diagnoses. The SAGE-SR was administered to healthy controls and outpatient mental health clinic clients to assess test duration and test-retest reliability. Cutoff scores for screening into follow-up diagnostic sections and criteria for inclusion of diagnoses in the differential diagnosis were evaluated.

**Results:**

The expert panel reduced the initial 1200 test items to 664 items that panel members agreed collectively represented the SCID items from the 8 targeted modules and DSM criteria for the covered diagnoses. These 664 items were iteratively submitted to 3 rounds of cognitive interviewing with 50 community mental health center participants; the expert panel reviewed session summaries and agreed on a final set of 661 clear and concise self-report items representing the desired criteria in the DSM-5. The SAGE-SR constructed from this item pool took an average of 14 min to complete in a nonclinical sample versus 24 min in a clinical sample. Responses to individual items can be combined to generate DSM criteria endorsements and differential diagnoses, as well as provide indices of individual symptom severity. Preliminary measures of test-retest reliability in a small, nonclinical sample were promising, with good to excellent reliability for screener items in 11 of 13 diagnostic screening modules (intraclass correlation coefficient [ICC] or kappa coefficients ranging from .60 to .90), with mania achieving fair test-retest reliability (ICC=.50) and other substance use endorsed too infrequently for analysis.

**Conclusions:**

The SAGE-SR is a computerized adaptive self-report instrument designed to provide rigorous differential diagnostic information to clinicians.

## Introduction

The Structured Clinical Interview for DSM-5 (SCID-5) is currently accepted as the gold standard in psychiatric diagnosis and is regularly used in research settings where the accurate diagnosis of primary and comorbid disorders is required for the appropriate determination of study eligibility and assignment to a research condition [1-3]. The SCID is also frequently used as the standard against which other diagnostic instruments are validated (eg, [4-8]). The structured format of the SCID with its direct adherence to Diagnostic and Statistical Manual of Mental Disorders (DSM) criteria accounts for its strong test-retest and inter-rater reliability for most diagnoses [1,2,3,9]. Overall, the full SCID-5-Research Version (RV) covers 63 diagnoses, takes an average of 90 min to administer, and requires considerable clinician training [2,10]. The Clinician Version (CV) of the SCID for DSM-5 (SCID-5-CV), released in 2014, consists of 10 modules that cover 39 of the most common diagnoses seen in clinical practice and allows screening for an additional 16 diagnoses [1]. Although it is easy to select individual SCID modules for administration, more complex customizations of items and diagnoses within modules can be difficult to implement.

To streamline use of the SCID in research and to make it more accessible for use in clinical settings, clinician-administered, Web-based versions of the SCID instruments were developed including the NetSCID-5-Clinician Version (NetSCID-5-CV), which covers the same disorders as the SCID-5-CV paper version; the NetSCID-5-Research Version (NetSCID-5-RV), which covers the same diagnostic modules of the paper version of the SCID-5-RV; and the NetSCID-5-Personality Disorder (PD) Version, which covers the 10 DSM-5 PDs across Clusters A, B, and C, as well as other specified PD [11]. In a validation study versus the paper version of the SCID-IV-RV, an earlier version of the NetSCID-RV demonstrated fewer data entry and branching errors than the paper version, was preferred by clinicians over the paper version, and was easier to administer [11]. In addition, anecdotal reports from clinicians indicate that NetSCID administration requires 30% less time than the corresponding paper SCID [11]. However, despite its advantages over the paper version of the SCID, the clinician NetSCID administration still requires significant clinician time and training, which may pose too large a burden for routine clinical care settings [12] and in epidemiological studies evaluating large numbers of participants where clinician-based interviewing becomes logistically prohibitive.

Perhaps primarily because of the need for time-efficient diagnostic practices, routine clinical practice continues to rely predominantly on unstructured clinical interviews [13], despite mounting evidence that doing so often results in missed comorbidities [14-16], missed diagnoses [17], and less-specific diagnoses (eg, adjustment disorders vs more specific mood or anxiety disorders) [15]. Some researchers suggest that clinicians who do not use structured interviews may sometimes narrow their diagnostic focus too quickly, thereby missing comorbid diagnoses, whereas structured interviews ensure clinicians assess a broader range of clinical diagnoses [3]. Research has shown that accurate diagnosis has implications for clients’ engagement in treatment and treatment outcomes [18], possibly linked to the role accurate diagnosis plays in the appropriate selection of evidence-based treatments [19]. The need for time-efficient and rigorous diagnostic practices is probably highest in primary care, where behavioral health problems are common presenting complaints and clinicians are under the highest pressure to assess and treat patients in a time-efficient manner [20].

Given the tension between the need for accurate diagnosis and the limited resource of clinician time in routine clinical practice, especially in primary care, one proposed solution is for patients to take a self-administered diagnostic screening questionnaire before their intake interview with a clinician. Results of this self-report screening measure could focus the clinician’s diagnostic expertise on a differential diagnosis during the face-to-face client interview [10,12,21], as well as satisfy the current mandate by the Affordable Care Act to routinely screen patients in primary care for depression and alcohol abuse [20]. To ensure that clinicians do not miss potential comorbidities, such screening questionnaires should be broad-based and cover a wide range of diagnoses frequently seen in clinical practice. Most currently available broad-based diagnostic screening measures either require a clinician or other trained interviewer to administer them (eg, Mini International Neuropsychiatric Interview–Clinician Rated, MINI-CR [7]; World Health Organization World Mental Health Composite International Diagnostic Interview, WHO WMH-CIDI [6,22]) or are only available with paper and pencil administration and scoring or do not correspond directly to DSM-5 criteria (eg, Clinical Interview Schedule–Revised [23-25]; Mini International Neuropsychiatric Interview–Patient Rated, MINI-PR [7]; the Psychiatric Diagnostic Screening Questionnaire [8]; Primary Care Evaluation of Mental Disorders [26]).

The goal of this study was to develop a computerized adaptive self-report assessment based on the SCID and DSM-5 criteria [27] complete with self-scoring and instantaneous report generation of a rigorous differential diagnosis for clinicians. Ideally, these reports would be immediately accessible through the client’s electronic health record. As with the NetSCID [11], the resulting assessment would be a HIPAA-compliant, Web-based software program that patients could complete at a mental health clinic or a primary care clinic using a desktop computer, laptop, tablet, or smartphone. Reports would enable clinicians to initiate a more focused routine diagnostic interview based on considerable background knowledge of the patient’s symptoms.

## Methods

### Stage I: Self-Report Item Pool Development

As a first step, we authored a set of approximately 1200 unique self-report items that mirrored the questions in the SCID for DSM-IV and corresponded with criteria outlined in the DSM-IV-TR. In anticipation of the release of DSM-5, we also developed items intended to represent the few anticipated changes to diagnostic criteria occurring between DSM-IV and DSM-5 (prospective changes were made available online before the DSM-5’s publication date). TeleSage staff developed these items using a rigorous methodology first developed and successfully implemented in our previous instrument development work [28]. Self-report items were drafted for 13 diagnostic categories judged to be the most commonly encountered in clinical practice by the developers of the SCID-CV [1]: (1) depressive disorders, (2) manic and hypomanic disorders, (3) generalized anxiety disorder (GAD), (4) panic disorder, (5) agoraphobia, (6) social anxiety disorder, (7) obsessive-compulsive disorder (OCD), (8) posttraumatic stress disorder (PTSD), (9) adult attention-deficit/hyperactivity disorder (ADHD), (10) psychotic disorders, (11) alcohol use disorder, (12) cannabis use disorder, and (13) other substance use disorders. Whenever the DSM included differing symptoms for “adolescents,” that wording was included as well to maximize the utility and flexibility of the resulting instrument.

During the item development process, staff members strove to keep items very simple by developing items that omitted lead phrases; omitted contingencies; included only a single concept; omitted idiomatic language; adhered to a 5-point Likert scale (never, rarely, sometimes, often, and always) wherever possible; used simple English language words; simple syntax, so as not to exceed a fifth grade reading level; and a consistent timeframe, where applicable, depending on the DSM-specified timeframe. These strategies were aimed at producing items that were easy to read, easy to understand, could be understood by non-native English speakers, and were amenable to direct translation into other languages. For SCID questions that were not straightforward (eg, questions that had multiple components), several simple self-report items were created. For example, to represent depression criteria 1A “In the last month, has there been a time when you were feeling hopeless, depressed, or down most of the day nearly every day,” 5 items were drafted: (1) I felt sad; (2) I felt depressed; (3) I felt irritable; (4) I felt hopeless; and a fifth item relating to “most of the day nearly every day.” We did not use the term “down” as it is idiomatic. We did create an item for hopelessness as it is in the DSM-5, although it is absent in DSM-IV-TR. We also created an item for irritability as it is a criterion for youth.

Given that we intended to develop readily understandable, clear expressions of clinical symptoms in simple language, we acknowledge that the content of some of our items may overlap with other existing measures. Indeed, 3 of the 5 items just referenced to represent DSM-5 depression criteria 1A are also present in the Patient-Reported Outcomes Measurement Information System (PROMIS) [29] item bank for depression. In developing their emotional distress items (within the domains of depression, anger, and anxiety), the PROMIS researchers, using an item response theory process, identified 78 different depression scales in the literature and found considerable overlap in the items covered [30]. In discussing the intellectual property rights regarding such items, these researchers noted that this overlap likely existed because the items “reflected generic aspects of emotional distress and the everyday language in which it is described” and thus “regarded them as part of the public domain because they reflected common-sense ideas about emotional distress” [30]. Our consultations with 2 lawyers specializing in intellectual property issues also supported the perspective that this is the case for simply worded individual items and small groups of items.

The completed item pool was iteratively reviewed by a panel of 7 experts, including Michael First, MD, the primary author of the SCID, 2 other psychiatrists, and 4 psychologists with combined expertise in community mental health, SCID items and administration, and mental health item development. Items were presented in tables populated with the original wording of the DSM criteria, the corresponding SCID item wording, and the proposed self-report items. The expert panel rated the clarity (1=unclear, 2=needs revision, and 3=clear) and correspondence with DSM-defined criteria (1=does not sufficiently correspond to DSM criteria, 2=needs to be rewritten to fully correspond to DSM criteria, and 3=directly and fully corresponds to DSM criteria) of each self-report item. Panel members also identified any missing concepts, offered suggestions for item rewrites, and discussed revised items by email and phone until consensus was reached on a final pool of items.

### Stage II: Cognitive Interviewing

The self-report item pool was divided into 2, with 6 to 7 diagnostic categories (approximately 4 SCID modules) in each half. After engaging in an institutional review board (IRB)-approved informed consent process, participants were given the half of the item pool that corresponded with their individual chart diagnosis. Both halves were then tested and revised over 3 rounds of cognitive interviewing (CI). After each round of CI, session summaries were analyzed by TeleSage staff. All items that posed difficulty for 20% or more of the participants were either omitted or rewritten for the next round of CI.

CI is a scientific technique that uses *verbal probes* and *verbal think alouds* to determine the perceived meaning of survey questions [31]. For this study, the cognitive interviewer presented each participant with a block of self-report items that corresponded to a single diagnostic category at a time. Item sets pertaining to each diagnostic category were presented in a balanced, randomized order to control for order effects and ensure that majority of the questions were completed.

After reading an item aloud, participants marked their responses to the items. In addition, participants were instructed to circle any item they perceived as unclear or confusing as they completed the self-report assessment. Participants were also encouraged to think aloud while they answered each item. After participants completed the self-report items, the cognitive interviewer asked follow-up questions to further assess the reason the participant found each circled item unclear or confusing, while also confirming that the participant understood the meaning and intent of items that were not circled. For example, the cognitive interviewer would point out specific words in the question and ask for the meaning of that word (eg, “Can you tell me what *irritable* means to you?”) or ask, for example, behaviors (eg, “You indicated that you “often” feel sad. Can you give me some examples of how you have felt sad in the past two weeks?”). This process continued until the interviewer probed all items. Interviews were recorded on a digital recorder, and the cognitive interviewer took objective, not interpretive, notes during the session pertaining to the participant’s responses as well. After the interview, the cognitive interviewer listened to the audio file as needed and converted the notes from the session into a summary indicating items that were particularly difficult for the participant to answer or caused confusion, and items for which the participant’s interpretation did not reflect the item’s intent. By having participants describe all their thoughts out loud as they work their way through questions, it is possible to identify many of the potential problems that could affect a patient’s response in unintended ways. Using CI to hone questions should improve the likelihood that individual items will ultimately have good psychometric characteristics during quantitative validation.

Each of the 3 rounds of CI was conducted with unique participants who engaged in an individual interview; no participant was interviewed twice. Participants in the first 2 rounds of CI were also given a clinician-administered SCID. This SCID contained the same modules (diagnostic categories) that the participants completed in the self-report item pool and included the participant’s specific chart diagnosis. To account for any learning effect, participants were randomized so that half of the participants took the SCID first and half completed the self-report items and CI first.

### Stage III: Screening Assessment for Guiding Evaluation-Self-Report Instrument Construction and Initial Validation

An expert panel was convened for this next stage to convert the self-report item pool into the computerized adaptive Screening Assessment for Guiding Evaluation-Self-Report (SAGE-SR). The panel included 2 psychiatrists, 2 clinical psychologists, 1 physician, TeleSage staff members with backgrounds in psychology as well as expertise in mental health item development and SCID administration, and TeleSage staff computer programmers with expertise in computer-adaptive instrument development. To construct an easily understood instrument that could be administered in a time-efficient manner, the SAGE-SR was constructed to have an initial 65-question screener which covered the same 13 diagnostic categories for which items were drafted in stage I. Respondents would need to endorse screener items at a sufficient threshold (set by the expert panel) within each diagnosis to “screen in” and branch to the remaining self-report items necessary to determine if respondents meet criteria for that diagnosis to be included in the final differential diagnosis. Possible diagnoses that could be returned in this differential diagnosis are presented in Table 1, along with the corresponding representation of diagnoses in the SCID-5-CV.

The expert panel examined the newly released DSM-5 criteria for each of the diagnoses covered by the self-report items to determine the most appropriate items for inclusion on the screener using clinical judgment for best fit and criteria that were “essential” or central to each diagnosis. For example, to meet DSM-5 criteria for major depressive disorder, 5 or more of a series of 9 symptoms must be present during the same 2-week period and represent a change from previous functioning [27]; however, 1 of these 5 symptoms must be either depressed mood or loss of interest or pleasure. Thus, the expert panel selected 3 self-report items for the screener to represent depressed mood (“I felt sad,” “I felt depressed,” and “I felt hopeless”) and 3 self-report items for the screener to represent loss of interest or pleasure (“I enjoyed life”—reverse coded, “I had difficulty enjoying things that I used to enjoy,” and “I was interested in my usual activities”—reverse coded). If a respondent met the threshold set by the expert panel on these screener items, the adaptive SAGE-SR would present the remaining depressive disorder items after the respondent completed the screener to determine if the respondent endorsed sufficient criteria for any depressive disorder to be considered for differential diagnosis. The expert panel also set the thresholds for determining whether respondents had endorsed sufficient criteria between the screener and follow-up questions for diagnoses to be reported for clinician consideration for differential diagnosis.

Once the initial instrument was constructed and programmed for Web-based administration (via personal computer, tablet, or smartphone), TeleSage staff members piloted and tested the Web-based administration of the SAGE-SR to identify any programming glitches. Following this process, healthy participants were recruited to take the SAGE-SR for the purpose of measuring administration time, assessing the appropriateness of the thresholds for screening and differential diagnosis set by the expert panel, identifying any remaining areas of confusion regarding item administration, and for preliminary quantitative validation. A subset of these participants returned for a second session within 1 week for the purpose of assessing test-retest reliability and how consistently participants screened into follow-up sections and received diagnoses for differential diagnostic consideration. All participants underwent a full informed consent process before engaging in any study procedures; all study and consent procedures were IRB-approved before the commencement of participant enrollment.

**Table 1:** Comparison of diagnoses covered by Screening Assessment for Guiding Evaluation-Self-Report and the Structured Clinical Interview for Diagnostic and Statistical Manual of Mental Disorders, Fifth Edition, Clinician Version.

DSM-5^a^ diagnostic category				SAGE-SR^b^ diagnoses or episodes covered	SCID-5-CV^c^ diagnoses or episodes covered^d^
**Mood disorders**					
	Major depressive episode			✓^e^	✓^f^
	Manic episode			✓^e^	✓^f^
	Hypomanic episode			✓^e^	✓^f^
	Persistent depressive disorder			✓	✓
	Major depressive disorder			✓	✓
	Other specified depressive disorder			✓	✓
	Bipolar I disorder			✓	✓
	Bipolar II disorder			✓	✓
	Other specified bipolar disorder			✓	✓
**Anxiety disorders**					
	Panic disorder			✓	✓
	Agoraphobia			✓	✓
	Social anxiety disorder			✓	✓
	Generalized anxiety disorder			✓	✓
	Other specified anxiety disorder				✓
	Anxiety disorder due to another medical condition				✓
	Substance/medication-induced anxiety disorder				✓
**Obsessive-compulsive and related disorders**								
	Obsessive-compulsive disorder			✓	✓
	Obsessive-compulsive and related disorder due to another medical condition				✓
	Substance/medication-induced obsessive-compulsive and related disorder				✓
**Trauma- and stressor-related disorders**								
	Posttraumatic stress disorder			✓	✓
**Neurodevelopmental disorders**								
	Attention-deficit/hyperactivity disorder			✓	✓
**Psychotic disorders**								
	Schizophrenia			✓	✓
	Schizophreniform disorder			✓	✓
	Schizoaffective disorder			✓	✓
	Delusional disorder			✓	✓
	Brief psychotic disorder			✓	✓
	Other specified psychotic disorder			✓	✓
	Psychotic disorder due to another medical condition				✓
	Substance/medication-induced psychotic disorder				✓
**Substance-related and addictive disorders**								
	Alcohol use disorder			✓	✓
	Cannabis use disorder			✓	✓
	Inhalant use disorder			✓	✓
	Other hallucinogen use disorder			✓	✓
	Opioid use disorder			✓	✓
	Phencyclidine use disorder			✓	✓
	Sedative, hypnotic, or anxiolytic use disorder			✓	✓
	Stimulant use disorder			✓	✓
	Other or unknown substance use disorder			✓	✓
Adjustment disorders					✓

^a^DSM-5: Diagnostic and Statistical Manual of Mental Disorders, Fifth Edition.

^b^SAGE-SR: Screening Assessment for Guiding Evaluation-Self-Report.

^c^SCID-5-CV: Clinician Version (CV) of the SCID for DSM-5.

^d^Screening questions are available on the SCID-5-CV for the following additional disorders: specific phobia, separation anxiety disorder, hoarding disorder, body dysmorphic disorder, trichotillomania, excoriation disorder, insomnia disorder, hypersomnolence disorder, anorexia nervosa, bulimia nervosa, binge eating disorder, avoidant/restrictive food intake disorder, somatic symptom disorder, illness anxiety disorder, intermittent explosive disorder, gambling disorder.

^e^Current episodes covered; past episodes under development.

^f^Current and past episodes covered.

 After completing the SAGE-SR, participants were asked to provide feedback on whether they found any items confusing or unclear, whether they found items repetitive, whether the instructions were clear, what they thought about the length of the assessment, how well the progress bar and radio buttons on the device functioned, as well as any other comments they wanted to offer on what worked and what did not.

In addition, an initial sample of respondents was recruited from an outpatient public sector mental health clinic based in Tennessee; initial results from this population were used primarily to examine administration time in a clinical population. Further recruitment at this site is ongoing and will be used for future quantitative validation in a clinical population.

## Results

### Stage I: Item Development

The expert panel iteratively rated, discussed, and rewrote items until each item scored on average 2.5 or higher for clarity and correspondence with its respective DSM symptom. A consensus was reached on a final pool of 664 items that expert panel members agreed were clear, easy to understand, and collectively represented the items from 8 SCID-IV-CV modules, thus covering the DSM-IV-TR criteria for each of 13 diagnostic categories (see Table 1 for diagnostic coverage).

### Stage II: Cognitive Interviewing

A total of 50 adult community mental health outpatients, including individuals with severe and persistent mental illness, were recruited from 2 locations at Centerstone, a private nonprofit mental health organization, in Nashville, TN, and in Bloomington, IN. Participants were recruited to ensure that they (according to their chart diagnoses) represented all 13 diagnostic categories in the self-report items (or 8 SCID-5-CV modules); participants ranged in age from 18 to 68 years (mean 39.9) and were 60% female (30/50), 86% white (43/50), 12% African American (6/50), and 2% Native American (1/50).

For the first round of CI, a total of 18 participants responded to approximately half of the final item pool of 664 items. Thus, each self-report item was tested in 9 cognitive interviews in the first round. After each interview, a staff member reviewed the recording of the interview and the cognitive interviewer’s notes from the session singling out the following: (1) items that were understood by everyone and (2) items that were difficult for some participants to answer or which were not interpreted as expected. Overall, by the end of the first round of testing, of the original 664 items, 157 items tested very well, 2 items were omitted, 1 item was split into 2 items, and small modifications were made to many additional items to increase clarity. Sample revised items are presented in Table 2, sample omitted items in Table 3, and sample retained items are presented in Table 4.

For the second round of CI, the 157 items that were understood very clearly were set aside, and 22 participants responded to approximately half of the remaining 506 unique items. Thus, each self-report item in the second round was tested in 11 more cognitive interviews. At the end of round 2, one more item was removed, and minor wording changes were made to several other items.

In the third round of CI, the 157 items that worked well in the first round were added back to the item pool to reassess the entire item pool. In addition, 10 CI sessions were conducted, each on half of the modules as before, so that each item received an additional 5 cognitive interviews. There were virtually no misunderstandings in this third round; less than 1% of items were described as confusing by any participant, and there was only 1 instance in which 2 people misunderstood the same item (this item had a content duplicate and was omitted).

**Table 2:** Examples of items revised during cognitive interviewing based on: participant think aloud and interviewer probing.

Sample revised items (with *intended diagnostic domain*)	Reason for revision
Original item: I felt anxious. Revised item: I had anxiety. *(Anxiety)*	Participants, particularly those in the South, sometimes defined anxious in the context of “I felt anxious” as excited or eager (eg, “I was anxious to go to the fair”). The noun form, however, did not have the same additional connotation; therefore, the item was revised to use the noun form of anxiety.
Original item: I thought I might be God’s personal messenger on Earth. Revised Item: I am the only person who can do God's work on Earth. *(Psychotic disorders-religious delusions* *)*	The original item produced a high base rate of endorsement among devoutly religious participants. The revised item is distinct from the notion that all people are God’s children or messengers.
Original instructions: Now I’m going to ask you about things you thought you might have seen while you were fully awake and it was light *.* Revised instructions: Now I’m going to ask you about things you might have seen while you were fully awake and there was enough light to see clearly. *(Psychotic disorders-visual hallucinations)*	Participant thinks aloud and interviewer probing responses indicated high endorsement because of the appearance of shadows due to dim light. The revised instructions clarify that visual hallucinations were present when enough light was present to see clearly (ie, eliminate shadows).

**Table 3:** Examples of items omitted during cognitive interviewing based on: participant think aloud and interviewer probing.

Sample omitted items (with *intended diagnostic domain*)	Reason for omission
I felt the presence of evil around me. *(Psychotic disorders–religious or persecutory delusion* *)*	Responses from participant think aloud and interviewer probing indicated that participants interpreted the item as meaning there were “bad people” (a bad element) around them, which led to a higher base rate of endorsement than was expected.
People said I did not show emotions. *(Psychotic disorders-affective flattening* *)*	Participants stated that people did not say this.

**Table 4:** Examples of items retained during cognitive interviewing based on: participant think aloud and interviewer probing.

Sample retained items (with *intended diagnostic domain*)	Reason for retaining item
I felt sad. *(Depression)*	Easily understood in early rounds of cognitive interviewing.
I had difficulty sitting still. *(Adult ADHD*^a^*)*	Easily understood in early rounds of cognitive interviewing .
I thought I deserved to be punished. *(Psychotic disorders-persecutory delusion or guilt)*	Easily understood in early rounds of cognitive interviewing and central to persecutory delusions.
I felt like my thoughts were being controlled against my will. *(Psychotic disorders-delusions of control* *)*	Easily understood in early rounds of cognitive interviewing and central to delusions of control.

^a^ADHD: attention deficit hyperactivity disorder.

On conclusion of all 3 rounds of cognitive interviews, the expert panel reviewed the session summaries and agreed on a final set of 661 items that they judged to be clear, concise, and that covered all 13 diagnostic categories. In general, the expert panel erred in keeping items that did well in CI, even if this made for some redundancy as expert panel members knew that the quantitative analysis would enable identification of the most predictive items and allow for future reduction of the item pool.

In the first and second rounds of CI, all 40 participants were also given a clinician-administered SCID. This SCID contained the same modules (and diagnostic categories) that the participants completed in the self-report item pool that included their specific chart diagnosis. The responses to all self-report items were compared with the same participant’s responses to the corresponding SCID item(s) to see whether the self-report items would predict the SCID response for the same item or symptom in a real-life application. In all the cases tested, we found that we could identify 1 or more self-report items that predicted each SCID item endorsement. More specifically, where participants selected 4 “often” or 5 “always” on the SAGE-SR (or in negatively scored items, a 1 “never” or 2 “rarely” on the Likert scale), the clinician independently endorsed the associated SCID item on the clinician-administered SCID.

### Stage III: Screening Assessment for Guiding Evaluation-Self-Report Instrument Construction and Initial Validation

Eighty-four participants who denied having sought treatment or received medication for a mental illness in the past two years were recruited in Chapel Hill, NC. To recruit participants, study staff passed out flyers describing the study near the campus of a large university and made calls to campus service organizations to describe the study; some participants were recruited directly by study staff through these efforts and others called in to schedule appointments when they learned about the study secondhand as a result of these recruitment strategies.

The resulting sample ranged in age from 18 to 34 (mean 20.2) years, was 74% female (62/84), 5% African American (4/84), 14% Asian (12/84), 7% Hispanic (6/84), and 68% white (57/84). An additional 5% of participants reported being of more than one race (4/84), and 1 participant declined to provide race information (1% or 1/84). All participants were asked to take the SAGE-SR using a tablet or laptop. A total of 42 participants returned within 7 days (mean 5.24 days) to take the SAGE-SR a second time. The 65-item screener covering 13 domains took an average of 7.3 min to administer to this nonclinical sample, with a standard deviation of 2.4 min. When the follow-up items were taken into consideration, the participants took an average 14 min to take the full SAGE-SR, with a standard deviation of 6.8 min. The Tennessee-based clinical sample was recruited via flyers posted in the clinic waiting room. This sample was comprised of 44 participants who ranged in age from 23 to 76 (mean 47.7) years and were 68% female (30/44). Race data was only available for 66% of this sample (29/44); of those that provided race information, the sample was 69% African American (20/29), 3% Asian (1/29), 14% Hispanic (4/29), 10% white (3/29), and 3% other (1/29). As expected, the screener took participants from the clinical sample longer to complete (average completion time of 9.4 min, with a standard deviation of 3.4 min). The full SAGE-SR took on average 24 min to administer in the public sector clinical sample, with a standard deviation of 12.6 min. In contrast, in research populations, the full NetSCID-CV takes 56 min to administer with a standard deviation of 34 min.

Feedback from the nonclinical sample indicated that participants found the SAGE-SR easy to navigate and complete and found nearly all items clear; one exception was the reference to “unwanted thoughts” in the section on obsessive-compulsive disorder, which participants indicated was too vague and confusing. To increase clarity, a definition was added to the display screen for this item: “Unwanted thoughts are thoughts that kept coming back to you even when you didn't want them to.” The only other feedback regarding clarity was regarding some lead prompts that were intended to prime participants to think of the particular period when they were experiencing the specific symptoms they endorsed during the screener to assess concurrence of the follow-up symptoms with the screener symptoms. For example, the lead prompt for the follow-up questions intended to explore generalized anxiety disorder initially read, “Because of my anxiety or worry,” but participants responded that reverse-scored questions did not work with this phrase; subsequently, the lead prompt phrase was changed to “During the time(s) when I felt anxious…” After this change, the related concurrency items were well understood.

The expert panel convened to review the results from the healthy sample to verify the appropriateness of the screening and diagnostic cutoff criteria. Relatively, few of the nonclinical participants were expected to screen in to take the follow-up questions, and fewer still were expected to meet criteria for inclusion of a diagnosis within the differential. Any items that were endorsed above threshold more than 15% of the time were reviewed by the expert panel. Thresholds for follow-up item administration were intended to be more sensitive, whereas thresholds for diagnosis were intended to be more specific. Minor threshold modifications were made after this review. For example, as mentioned earlier, 3 self-report items represented depressed mood on the screener (“I felt sad,” “I felt depressed,” and “I felt hopeless”); initially, the threshold for screening in to the follow-up depression items was endorsing any of these 3 items as happening at least “sometimes” in the last 30 days. This threshold worked well for the “I felt depressed” and “I felt hopeless” items but was overinclusive for the “I felt sad” item (too many participants screened in), so the screening threshold for that item was changed to at least “often.” In addition, when looking at the consistency with which participants screened in to receive depression follow-up questions, participants who only screened in at 1 time point did so by answering the “I felt sad” screener question at the “sometimes” threshold at that time point; therefore, increasing the threshold for this item also increased the consistency of the screening algorithm.

As part of our preliminary look at quantitative validation, test-retest reliability estimates were calculated for the screening items that were always administered in each of the 13 diagnostic categories covered by the SAGE-SR in the nonclinical sample (the screener section also includes some branching, so all items were not answered by every participant). For the 8 screening modules where the initial screener items included only Likert scale items, we first calculated summary scores within each module and then calculated the intraclass correlation coefficient (ICC) for these summary scores. The screening module for psychotic disorders includes Likert scale items that could indicate hallucinations as well as delusions, so summary scores and ICCs were also calculated for these subcategories of the psychotic disorders screening module. The ICC model used for these analyses was a 2-way mixed model of absolute agreement because the rater was the same at test and retest (self-report). This ICC model was also used to calculate test-retest reliability for the alcohol use disorders screening item, which was a continuous measure of the number of days the participant drank alcohol in the last 30 days. For the remaining 4 modules, categorical items (answered either yes or no) were used for screening purposes (for panic disorder, participants were asked whether they had ever had a panic attack, whereas, for both cannabis and other substance disorders, participants were asked whether they had any use within the past 30 days. For PTSD, participants were asked 4 questions about whether they had (1) ever experienced serious trauma, (2) witnessed serious trauma, (3) had a close friend or relative who was traumatized, or (4) whether they were repeatedly exposed to trauma through their work). For each of these 7 items, we calculated kappa coefficients as a measure of test-retest reliability; however, it was not possible to calculate a kappa coefficient for the diagnostic screening module for other substance use disorders, given that only 1 individual endorsed use in the past 30 days and did so at both time points, leaving empty cells and constants in the 2-way tables. The remaining test-retest reliability results are presented with 2-tailed 95% CIs (using bootstrap methods for the kappa coefficients) in Table 5.

**Table 5:** Test-retest reliability of 12 diagnostic screening modules of the Screening Assessment for Guiding Evaluation-Self-Report.

Diagnostic screening module		Test-retest reliability	95% CI	*P* value
Depressive disorders^a^		.67	0.46-0.81	<.001
Manic and hypomanic disorders^a^		.50	0.23-0.70	<.001
Generalized anxiety disorder^a^		.60	0.29-0.77	<.001
Panic disorder^b^		.86	0.67-1.00	<.001
Agoraphobia^a,c^		.90	0.82-0.94	<.001
Social anxiety disorder^a^		.83	0.70-0.91	<.001
Obsessive–compulsive disorder^a^		.68	0.33-0.85	<.001
Posttraumatic stress disorder^b^–ever experienced serious trauma		.86	0.63-1.00	<.001
Posttraumatic stress disorder^b^–ever witnessed serious trauma		.60	0.22-0.90	<.001
Posttraumatic stress disorder^b^–close family member or friend experienced serious trauma		.76	0.55-0.95	<.001
Posttraumatic stress disorder^b^–repeated exposure to traumatic events through work		.79	N/A^d,e^	<.001
Adult attention-deficit/hyperactivity disorder^a^		.63	0.27-0.82	<.001
**Psychotic disorders** ^a^		.72	0.41-0.86	<.001
	Hallucinations^a^	.65	0.44-0.80	<.001
	Delusions^a^	.74	0.31-0.89	<.001
Alcohol use disorder^a^		.70	0.50-0.82	<.001
Cannabis use disorder^b^		.84	0.64-1.00	<.001

^a^Test-retest reliability measure is an intraclass correlation coefficient (2-way mixed model of absolute agreement).

^b^Test-retest reliability measure is a kappa coefficient.

^c^The distribution of summary scores in the agoraphobia domain was highly skewed; a log transformation was performed before calculating the intraclass correlation coefficient for this domain.

^d^N/A: not applicable.

^e^Bootstrap methods were unsuccessful to generate a confidence interval for the kappa coefficient for the posttraumatic stress disorder screening question regarding exposure to trauma through work because of the low base rate of this occurrence in our primarily college student sample.

In determining how to interpret these measures of reliability, we used 2 relevant resources: (1) the presented rationale for interpreting the reliability coefficients used by the researchers conducting the DSM-5 field trials [32,33] and (2) the similar ranges or rationale suggested by Cicchetti [34]. In each of these resources, scores below .60 are considered “fair” or “questionable.” Scores from .60 to .75 [34] or .80 [32,33] are considered “good,” whereas scores above either .75 or .80 are considered “excellent.” Within this framework, test-retest reliabilities for agoraphobia, social anxiety disorder, cannabis use disorder, panic disorder, and 1 (to 3, depending on whether the. 75 or .80 range endpoint is used) of the PTSD items were “excellent,” whereas those for depression, GAD, OCD, ADHD, one (to 3) of the PTSD items, psychotic disorders, and the subdomains of hallucinations and delusions were “good.” The only domain to not reach at least “good” for test-retest reliability was mania or hypomania, which is consistent with previous attempts to develop self-report items for this diagnostic category [7,8,33].

## Discussion

### Principal Findings

The SAGE-SR was developed as a self-report alternative to the SCID and NetSCID-CV. The development process included the use of an expert panel to draft and iteratively review items as well as review the results of CI regarding item clarity to ensure that the criteria for 13 diagnostic categories commonly seen in clinical practice were well represented in a final pool of 661 well-understood self-report items. Using this item pool, we constructed the SAGE-SR as a 2-part computerized adaptive assessment with an initial 65-item screening instrument from which respondents who meet screening thresholds branch to follow-up questions to determine which diagnoses are returned for a clinician to consider for differential diagnosis.

Initial validation efforts with a nonclinical sample yielded promising results; qualitative feedback from participants indicated items and instructions were well understood, whereas the tablet- or laptop-based administration was simple to complete and reasonable in length. Preliminary quantitative validation efforts suggest good consistency in screening algorithms across 2 administration times as well as good to excellent test-retest reliability across all but 1 diagnostic category for the screening items in our small nonclinical sample. The one domain for which test-retest reliability was weakest was mania or hypomania, which has also proven problematic for other researchers attempting to create self-report diagnostic screening assessments [7,8,33]. The expert panel made minor revisions to the mania or hypomania self-report items and screening algorithms; whether these revisions improve the test-retest reliability of these items will be addressed in the results from the ongoing quantitative validation with a larger clinical sample.

### Limitations

We believe that the item development and qualitative validation procedures described above were very comprehensive, but although the initial quantitative feedback indicates that the SAGE-SR has great promise, the quantitative results are preliminary and based on a small nonclinical sample. Clearly, the results of this initial validation study will need further replication in a larger clinical sample. Data collection in clinical samples is ongoing, and more extensive quantitative validation will be presented once that work is complete. In addition, as noted earlier, the SCID is typically the gold standard against which the accuracy of most diagnostic assessments is measured. A cross-validation of the SAGE-SR’s differential diagnosis against the NetSCID-5-CV’s diagnostic algorithms is also currently underway.

### Conclusions

The SAGE-SR has an initial diagnostic screener that branches to groups of follow-up items to efficiently produce a differential diagnosis. Because the assessment is self-report, it should be possible to use the SAGE-SR in routine clinical care both in specialty behavioral health and in primary care settings. The SAGE-SR offers the promise of providing a rigorous differential diagnosis based on the SCID-5-CV and DSM-5 to a clinician before their meeting with the client so that their face-to-face time can be focused on clarifying that diagnosis in a manner that builds the rapport so inherent in the success of a therapeutic relationship. Indeed, an additional critique offered against the use of either the SCID-5 or other structured clinical interviews in clinical settings is that, despite the diagnostic rigor they provide, it is difficult to build rapport while adhering to a strict and standardized administration protocol [10].

The SAGE-SR helps address the concerns in the field regarding the need for greater diagnostic rigor as well as assessment of possible comorbidities that might be missed in unstructured clinical interviews while doing so in a cost-effective and clinician time-effective manner. The SAGE-SR also fits into the health care movement exemplified by the personal health record in which patients are empowered to provide information to their clinicians and to participate more actively in determining what treatment is most appropriate for them. The SAGE-SR could help primary care practices satisfy the Affordable Care Act’s mandate for screening for depression and alcohol use, while doing so as part of a more comprehensive screen for common behavioral health issues.

In addition to its utility for use in routine clinical care in primary care and specialty behavioral health settings, the SAGE-SR offers rigorous coverage of disorders and utility to clinical researchers as well as for epidemiological studies evaluating large number of participants where clinician-based interviewing is not feasible or is prohibitively expensive. The SAGE-SR covers the same diagnostic categories as the SCID-5-CV and all clinical diagnoses in these categories except for psychiatric diagnoses due to another medical condition and substance-induced diagnoses (see Table 1). Thus, the SAGE-SR covers 28 of the 35 disorders in the 8 primary modules of the SCID-5-CV while taking approximately half as long for respondents to complete and without the training and administration time burdens for the clinician. Like the NetSCID-5-CV, responses to the SAGE-SR populate a detailed database but, unlike the NetSCID-5-CV, the SAGE-SR gathers much more information that could then be available for quantitative analysis. Rather than generating a series of binary criteria endorsements, the SAGE-SR generates a very granular and complete inventory of individual symptoms with Likert scale frequency assessments, thus offering both diagnostic and symptom severity information. This detailed electronic response set can be used to populate admission summaries, progress notes, and discharge summaries, as well as offer a wealth of information on treatment progress and response. The detailed database from the SAGE-SR responses over time can be used to identify the symptom clusters that respond best to specific interventions and maximize the likelihood of measuring change quantitatively to be able to identify best practices.

Given the move toward measurement-based care [35-37], the information provided by the SAGE-SR can potentially be used to look at symptom presentation and severity across multiple time points as well as help clinicians monitor cross-cutting symptoms that might not be part of a primary diagnosis to help justify diagnostic and treatment decisions, fulfilling one of the recommendations of the DSM-5 [33]. In the future, it should also be possible to rescreen clients with the most important items. For example, if OCD, panic disorder, and major depressive episode are included in the differential diagnosis, then the corresponding self-report Likert scale items could be administered at regular intervals. This very focused approach to outcomes tracking should minimize clinician and patient burden. Thus, the SAGE-SR represents a potentially invaluable tool in the move toward measurement-based care.

More information about the SAGE-SR is available on the Web [38] as is a demonstration version of the SAGE-SR [39].

## Abbreviations

ADHD: attention deficit hyperactivity disorder
CI: cognitive interviewing
CIDI: Composite International Diagnostic Interview
CV: Clinician Version
DSM: Diagnostic and Statistical Manual of Mental Disorders
GAD: generalized anxiety disorder
ICC: intraclass correlation coefficient
IRB: institutional review board
MINI: Mini International Neuropsychiatric Interview
OCD: obsessive-compulsive disorder
PD: personality disorders
PROMIS: Patient-Reported Outcomes Measurement Information System
PTSD: posttraumatic stress disorder
RV: Research Version
SAGE-SR: Screening Assessment for Guiding Evaluation-Self-Report
SCID: Structured Clinical Interview for DSM
WHO WMH-CIDI: World Health Organization World Mental Health Composite International Diagnostic Interview
